# Medical Ultrasound Video Coding with H.265/HEVC Based on ROI Extraction

**DOI:** 10.1371/journal.pone.0165698

**Published:** 2016-11-04

**Authors:** Yueying Wu, Pengyu Liu, Yuan Gao, Kebin Jia

**Affiliations:** 1 Beijing Advanced Innovation Center for Future Internet Technology, Beijing University of Technology, Beijing, China; 2 Beijing Laboratory of Advanced Information Networks, Beijing, China; 3 College of Electronic Information and Control Engineering, Beijing University of Technology, Beijing, China; Shenzhen institutes of advanced technology, CHINA

## Abstract

High-efficiency video compression technology is of primary importance to the storage and transmission of digital medical video in modern medical communication systems. To further improve the compression performance of medical ultrasound video, two innovative technologies based on diagnostic region-of-interest (ROI) extraction using the high efficiency video coding (H.265/HEVC) standard are presented in this paper. First, an effective ROI extraction algorithm based on image textural features is proposed to strengthen the applicability of ROI detection results in the H.265/HEVC quad-tree coding structure. Second, a hierarchical coding method based on transform coefficient adjustment and a quantization parameter (QP) selection process is designed to implement the otherness encoding for ROIs and non-ROIs. Experimental results demonstrate that the proposed optimization strategy significantly improves the coding performance by achieving a BD-BR reduction of 13.52% and a BD-PSNR gain of 1.16 dB on average compared to H.265/HEVC (HM15.0). The proposed medical video coding algorithm is expected to satisfy low bit-rate compression requirements for modern medical communication systems.

## I. Introduction

With the proliferation of digitalization processes in modern hospitals, digital medical video plays an increasingly important role in the diagnosis and treatment of diseases. Large amounts of medical data are produced in the widespread application of digital medical video, resulting in a serious challenge in terms of its storage and transmission. However, medical communication systems [1] based on JPEG or JPEG2000 standards have difficulty in satisfying the requirements of medical video compression. Therefore, it is of great practical significance to research high-efficiency medical video compression techniques.

The high efficiency video coding (H.265/HEVC) standard [2], which was established and developed by the Joint Collaborative Team on Video Coding (JCT-VC), can achieve a 61.63% and 20.26% reduction in coding bit-rate requirements with equal objective quality compared to the JPEG and JPEG2000 standards for intra compression mode, respectively [3]. Given the growing demand for medical video coding efficiency and the excellent performance improvement of H.265/HEVC encoders, a medical video coding algorithm based on H.265/HEVC has drawn considerable research attention. Initially, Panayides et al. [4] integrated H.265/HEVC with the Mobile Health (M-health) Communication System and improved coding performance while maintaining acceptable diagnostic visual quality for medical video. Based on this work, Panayides et al. [5] further used H.265/HEVC to encode ultrasound video and verified the coding capacity of H.265/HEVC in medical applications. Shenthil et al. [6] improved the coding performance by incorporating modified sample adaptive offset (SAO) into medical video compression based on H.265/HEVC. As reported in [7], Panayides et al. used a pre-filtering procedure prior to medical video coding in the H.265/HEVC framework. A series of developments was also proposed to better address the new challenges found in the medical video coding domain [8]. Two medical sequences (namely, CT_LongrunShort and CT_Cardiac) were added to the H.265/HEVC test sequences. In general, this research has provided a substantial theoretical foundation and feasible support for the application of the H.265/HEVC standard in the digital medical video coding domain.

In the above algorithms, the H.265/HEVC standard was directly applied only to the medical video compression domain; rarely were the characteristics of digital medical images taken into account. However, the neglected characteristics of digital medical video can also provide a favorable opportunity to further remove redundant data for video coding standards.

**Fig 1:**
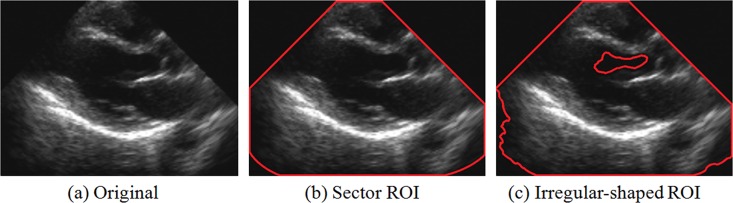
Examples of traditional ROIs (a) Original, (b) Sector ROI, and (c) Irregularly shaped ROI.

Only a few segments of medical video are associated with clinical diagnoses; these are referred to as diagnostic-regions-of-interest (d-ROIs, hereinafter referred to as ROIs). In this context, ROI-based medical video hierarchical coding technologies with respect to the JPEG standard have been thoroughly studied. For instance, Sridhar [9] and Moorthi et al. [10] allocated different encoding strategies for ROIs and non-ROIs under the JPEG coding framework to reduce the coding bit-rate of medical video. However, the ROI extraction results in traditional algorithms were usually defined as regularly shaped regions (e.g., rectangles or sectors) or irregularly shaped regions (which overlapped with the edge of human tissues and organs) by artificial selection. Fig 1 shows examples of ROI descriptions using traditional methods. This limitation might constitute a serious obstacle for special quad-tree coding structures when the traditionally shaped ROIs are directly used in the H.265/HEVC standard. Therefore, it is of great significance to design a hierarchical coding mechanism based on a properly shaped ROI extraction strategy for medical video compression.

To reduce the coding bit-rate while maintaining acceptable visual quality, two key techniques in medical video compression with respect to ROI extraction and hierarchical coding are presented in this paper. First, a ROI extraction strategy based on the H.265/HEVC quad-tree structure is introduced. Second, a hierarchical coding scheme based on transform coefficient adjustment and quantization parameter (QP) selection is designed.

The remainder of this paper is organized as follows. Section II and section III, respectively provide techniques for ROI extraction and hierarchical coding in detail. Section IV presents extensive experimental results and discussion to verify the coding performance of the proposed method. Finally, section V presents conclusions.

## II. ROI Extraction with Quad-tree Structure

An effective and rapid ROI extraction strategy, which can be appropriate for both the characteristics of medical images and the special coding structure in H.265/HEVC, is the foundation for optimizing coding performance.

According to the human visual selective attention mechanism, prominent texture regions might attract more attention than sparse texture regions. For medical video in particular, quantitative and qualitative changes in texture information often reflect the pathologic variations of disease [11] and also provide a crucial reference for clinical diagnoses. Therefore, textural information can be an important principle for distinguishing ROIs and non-ROIs.

### A. Texture Feature Vector Selection

Choosing parameters to describe the texture information of medical images is the key step for ROI detection. In the field of digital image processing, statistical features, including the **mean**, the **standard deviation**, and the **entropy**, are commonly used to describe the textural information of current images or regions.

First, suppose *I*(*i*,*j*) is the pixel value of the current region at position (*i*,*j*) and *N* × *N* represents the size of the current region. Then, the physical significance and calculation of the following three textural features are as follows.

- The ***mean* (*μ*)** represents the average pixel value in the current region. For medical images, the non-ROIs appear as essentially black areas, so *μ* has a smaller value.
  $$\mu =\frac{1}{N^{2}}\sum_{i=1}^{N}\sum_{j=1}^{N}I\left(i,j\right);$$ (1)
- The ***standard deviation* (*σ*)** represents the dispersion of pixel values in the current region. For medical images, non-ROIs are relatively homogeneous, so *σ* has a smaller value.
  $$\sigma^{2}=\frac{1}{N^{2}}{\sum_{i=1}^{N}\sum_{j=1}^{N}I\left(i,j-\mu \right)}^{2};$$ (2)
- The ***entropy* (*H*)** reflects the uncertainty of information distribution in the current region. For medical images, there is little information in non-ROIs, so *H* has a lower value.
  $$H=-\sum_{i=0}^{255}p_{i}log\left(p_{i}\right);$$ (3)
  where *p*_*i*_ represents the proportion of pixel values *i* in the current region.

**Fig 2:**
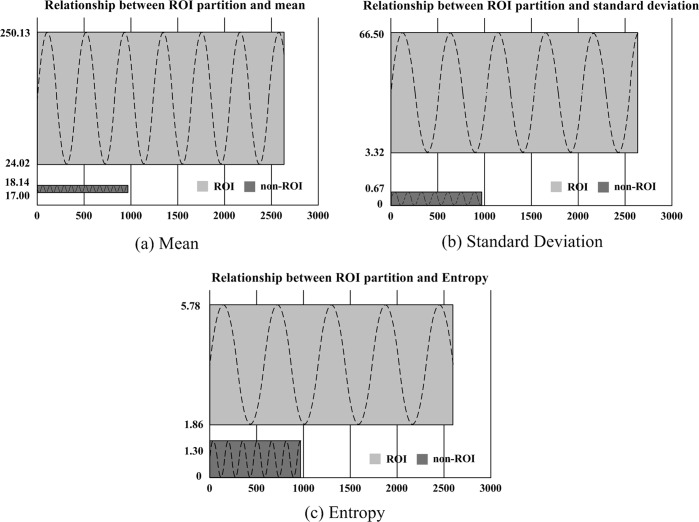
Relationship between ROI partition and (a) mean, (b) standard deviation, and (c) entropy.

Second, the effectiveness in ROI extraction using the three textural features can also be demonstrated by the experimental results. The *μ*, *σ*, and *H* of 3,640 8×8 pixel regions (including 2,664 ROIs and 976 non-ROIs) were calculated. Fig 2 shows the relationships between the three textural features and the ROI partitions.

As shown in Fig 2, the statistical results illustrate that the values of ***μ***, ***σ***, and ***H*** were concentrated in the intervals of [17.0000 18.1406], [0 0.6659], and [0 1.3019], respectively, for non-ROIs. The corresponding values were widely distributed in the intervals of [24.0156 250.1250], [3.0249 66.4984], and [1.8552 5.7813], respectively, for ROIs. Therefore, there was a significant distinction in the distribution of ***μ***, ***σ***, and ***H***, which provides effective evidence for the segmentation of ROIs and non-ROIs.

In another experiment, the 3,640 8×8 pixel regions were divided into two groups. Each group contained 1,332 ROIs and 488 non-ROIs. The textural feature vectors *T* were defined as *T* = [*μ*, *σ*, *H*]. *T* of the first group was used as a training set, and *T* of the second group was used as a test set. Then, the two groups of *T* were introduced to the nearest neighbor (NN) classifier [12] with a pre-normalization process for the purpose of verifying the classification effect. The experimental results indicated that the classification accuracy of the test set was 92.83%. Compared to the high-dimensional textural description vectors, such as the gray level co-occurrence matrix (GLCM) [13] and the local binary pattern (LBP) [14], the proposed textural feature vectors reduced the pre-process computational burden and guaranteed the precision of ROI extraction.

### B. Quad-tree Shaped ROI Extraction

Based on the above experimental findings, *T* = [*μ*, *σ*, *H*] was used to describe the textural information in the current coding units (CUs). Then, the calculation and classification procedure of *T* was as follows.

First, the to-be-encoded CU was segmented into the smallest coding unit (SCU) with a size of 8×8.

Second, supposing *SCU*(*i*) represents the *i*-th SCU of the to-be-encoded CU, then the texture feature vector *T*_*SCU*_ (*i*) = [*μ*, *σ*, *H*] of the current SCU was calculated according to formulae (1)—(3).

Finally, the normalized *T*_*SCU*_ (*i*) was sent to the NN classifier for calculating the category *C*(*SCU*(*i*)) of the current *SCU*(*i*).

The above process achieved the classification results of all previous SCUs in the to-be-encoded CU.

Further, the category *C*(*CU*) of the CU was derived by the classification result of each SCU according to formula (4). Suppose 2*N*×2*N* represents the size of CU, where *N∈* {4,8,16,32}. ║ ║ represents the process for obtaining the number of the elements in the current set. Then, the *C*(*CU*) was calculated by

$$C\left(CU\right)=\left\{ \begin{array}{l} ROI,\;if\;8\times 8\times \left\|i|C\left(SCU\left(i\right)\right)=ROI\right\|/\left(2N\times 2N\right)\geq 1/4\\ non-ROI,\;if\;8\times 8\times \left\|i|C\left(SCU\left(i\right)\right)=ROI\right\|/\left(2N\times 2N\right)<1/4 \end{array}\right.;$$ (4)

**Fig 3:**
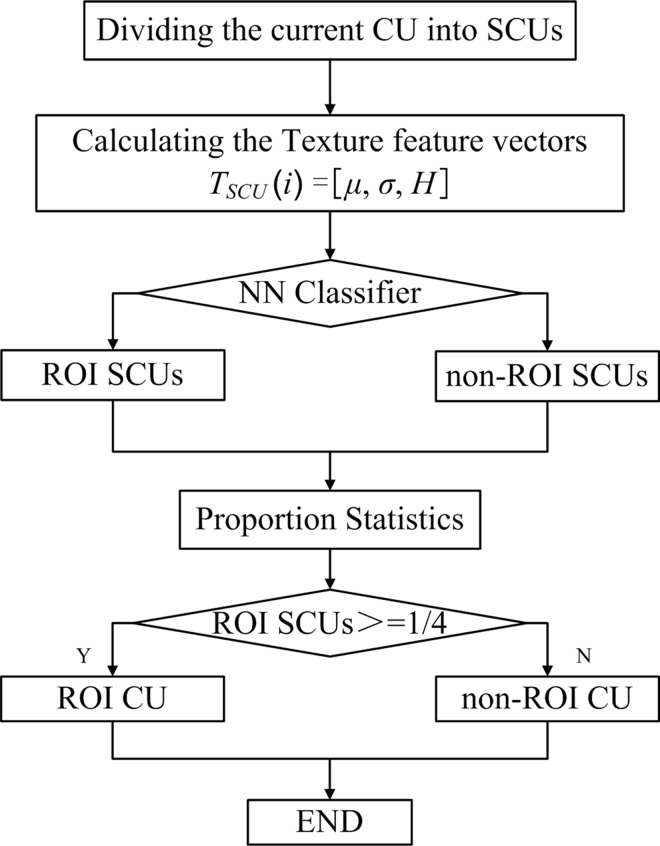
ROI CU extraction framework.

Thus, if the proportion of ROI SCUs was < 1/4, the to-be-encoded CU will be categorized as non-ROI. Otherwise, the to-be-encoded CU will be categorized as ROI. The ROI CU extraction framework is illustrated in Fig 3.

**Fig 4:**
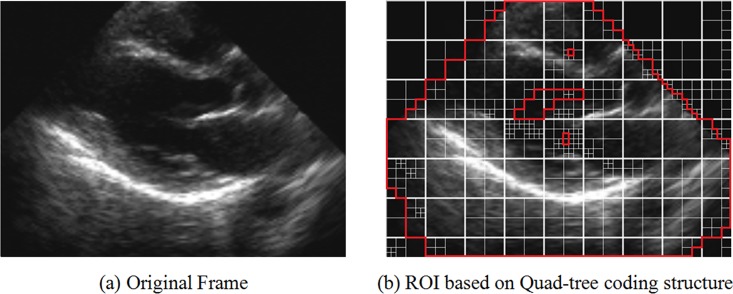
ROI extraction result based on HEVC quad-tree coding structure (a) original frame, (b) ROI based on quad-tree coding structure.

As described in the ROI extraction framework, it can be observed that the ROIs were composed of every ROI CU in the proposed ROI extraction method. Compared to the traditional ROIs shown in Fig 1, the proposed ROI extraction strategy may be more suitable to the flexible quad-tree coding structure in H.265/HEVC and provide convenient implementation of the subsequent hierarchical coding process. Fig 4 shows the ROI extraction result based on the H.265/HEVC quad-tree coding structure.

## III. Hierarchical Coding

The hierarchical compression scheme is the specific implementation procedure for reducing the coding bit-rate while maintaining acceptable visual quality.

Transform and quantization process is the most important part of the H.265/HEVC standard, which can remove data redundancy by transforming and quantifying the prediction residuals. Meanwhile, the quality of the reconstructed video and the coding bit-rate are directly related to the transform and quantization process. Therefore, for reduction of the coding bit-rate and improvement of the visual quality, a hierarchical coding scheme is proposed in this paper with respect to the transform and quantization process in the H.265/HEVC coding framework.

### A. Transform Coefficient Adjustment

The transform coefficient is the concrete manifestation of image pixel information in the transform domain. The value of the transform coefficient directly reflects the overall distribution of image brightness and energy and contains the details of image texture and edges. Therefore, it can be concluded that the transform coefficient is of important significance to the visual quality of reconstructed medical video. However, the discrete cosine transform (DCT) and discrete sine transform (DST) with fixed transform matrices are used to calculate the transform coefficients of the prediction error residual in the H.265/HEVC standard. A number of transform coefficients with smaller values are set to zero after the quantization process. This approach may lead to the loss of image details and insufficient visual contrast. Therefore, to selectively highlight the textural information in medical video, the idea of frequency-domain-based image enhancement is introduced to the transform coding of prediction residuals in H.265/HEVC.

On this basis, a transform coefficient equilibrium matrix was used to adjust the transform coefficients of the prediction residual. The specific procedure was as follows.

First, the texture density parameter *T*_*D*_ was defined to describe the variation degree of the textural information in medical video. Additionally, the *T*_*D*_ value was computed according to the previous ROI extraction results and the intra perdition mode in H.265/HEVC:

$$T_{D}=\left\{ \begin{array}{l} 2,\;if\;C\left(CU\right)\in ROI,\;\text{and}\;Intra\;Mode\in Intra\;Angular\\ 1,\;if\;C\left(CU\right)\in ROI,\;\text{and}\;Intra\;Mode\in Intra\;DC\;\text{or}\;Intra\;Planar\\ 0,\;if\;C\left(CU\right)\in non-ROI \end{array}\right.;$$ (5)

where *Intra Mode* represents the intra prediction mode of the current prediction unit (PU). For ROIs, if the intra prediction mode belongs to the angular mode, it can be assumed that there is directional-variation texture information in the current PU, and *T*_*D*_ = 2; otherwise, if the intra prediction mode belongs to the planar or DC mode, it can be assumed that there are slow-variation texture features in the current PU, and *T*_*D*_ = 1. For non-ROIs, *T*_*D*_ = 0.

Second, the transform coefficients were adjusted by the transform coefficient equilibrium matrix Ω, which was defined as follows:

$$\Omega ={\left(m_{ij}\right)}_{n\times n}=\left[\begin{array}{ccc} m_{11} & \cdots & m_{1n}\\ \vdots & \ddots & \vdots \\ m_{n1} & \cdots & m_{nn} \end{array}\right],\;n\in \left\{4,8,16,32\right\};$$ (6)

where the matrix element *m*_*ij*_ of the current transform coefficient equilibrium matrix Ω was defined as follows according to texture density parameter *T*_*D*_, where *i* and j, respectively represented the row and column position of the matrix element *m*_*ij*_:

$$m_{ij}=\{\begin{array}{l} -0.05T_{D},\;if\;C\left(CU\right)\in ROI\\ 0.1,\;if\;C\left(CU\right)\in non-ROI \end{array},\;T_{D}\in \left\{0,1,2\right\};$$ (7)

During the transform coefficient adjustment process, if *T*_*D*_ = 0, the transform coefficients were reduced. Otherwise, the transform coefficients were increased if *T*_*D*_ = 1 or 2.

Finally, based on Ω, the transform coefficient adjustment process is described as follows:

$$\hat{H}=\left\lfloor H⊗\left|1-\Omega \right|\right\rfloor ;$$ (8)

where *Ĥ* represents the transform coefficients after the adjustment process and *H* denotes the transform coefficients calculated by the H.265/HEVC standard.

This approach indirectly enhances the contrast of texture-significant regions in medical video by the revision of the transform coefficients in the frequency domain. This revision can maintain the reliability and accuracy of medical video for clinical diagnosis and disease treatment.

### B. QP Selection

The visual quality and compression ratio of reconstructed video are closely related to the selection of the QPs in the H.265/HEVC standard. Smaller QP values result in higher reconstructed video quality and lower compression efficiency, and vice versa. Thus, to guarantee the visual quality of ROIs and reduce the associated coding bit-rate cost, an ordinary QP value was selected (as in [7]) to encode the transform coefficients of ROIs in medical video after the transform process, while the QP value in non-ROIs was equal to

$$QP_{non-ROI}=QP_{ROI}+△QP$$ (9)

In formula (9), the value of △*QP* can range from 10 to (51-*QP*_*ROI*_). The coding bit-rate decreases with increasing △*QP*.

**Fig 5:**
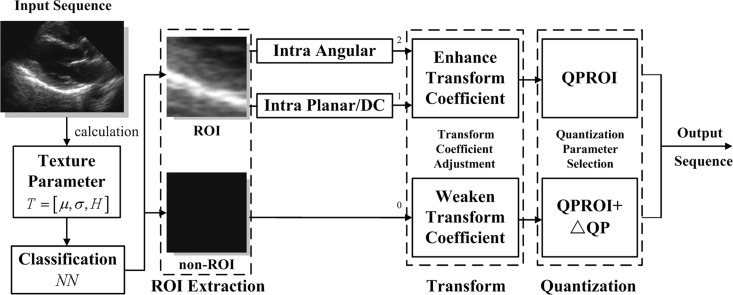
Hierarchical coding framework of the proposed method.

Fig 5 shows the integrated hierarchical coding framework based on the two key techniques of transform coefficient adjustment and QP selection.

## IV. Results and Discussion

The experimental environment and results are described as follows.

### A. Experimental Configuration

#### a) Encoder Configuration

To validate the coding performance of the proposed method, gain or loss was measured with respect to the H.265/HEVC test model (HM15.0) under all intra-coding modes. The specific encoder configuration is summarized in Table 1. The maximum CU width and height and the CU partition depth were set to 64 and 4, respectively. Novel coding tools introduced in H.265/HEVC, including transform skip (TS), sample adaptive offset (SAO), and wave-front parallel processing (WPP), were enabled.

**Table 1:** H.265/HEVC encoding parameters and coding tool configuration.

Parameter	Value	Coding Tools	ON/OFF
Max CU Height and Width	64	Transform Skip	ON
CU Partition Depth	4	Sample Adaptive Offset	ON
Intra-period/GOP Size	1	Wave-front Parallel Processing	ON

#### b) Experimental Dataset

The coding performance was characterized using different medical ultrasound video with a spatial resolution of 560×416 and 640×480 at 30 frames per second (fps). The medical ultrasound videos used in this paper can be accessed from the MedPix Database [15], which is supported by the USUHS department. For each video, four different coding bit-rate values were generated by varying the QPs at 22, 27, 32, and 37, and △*QP* was set to 10.

### B. Experimental Result and Performance Analysis

The experimental results and performance analysis are stated below in terms of bit-rate saving, rate-distortion (R-D) performance, and video quality.

#### a) In terms of bit-rate savings

For demonstrating the superiority in terms of bit-rate savings, the statistical results in Table 2 show the coding bit-rate consumption of the proposed hierarchical medical video coding strategy compared to the original H.265/HEVC (HM15.0) algorithm and the H.265/HEVC algorithm combined with the modified SAO process in [6].

**Table 2:** Comparison of the coding bit-rate with the H.265/HEVC standard.

Sequence	QP	HM15.0	[6]		Proposed	
(Resolution)		Bit-rate	Bit-rate	△Bit-rate	Bit-rate	△Bit-rate
		(Kbps)	(Kbps)	(%)	(Kbps)	(%)
**Ultrasound video**	22	3425.800	3402.600	-0.68	2973.240	-13.20
	27	2220.100	2206.850	-0.60	1908.680	-14.03
	32	1386.750	1374.300	-0.90	1160.040	-16.35
**(560×416)**	37	817.150	805.650	-1.41	671.720	-17.80
	Average results					
	QP	-	-	-0.90	-	-15.35
**Ultrasound video**	22	8962.500	8914.600	-0.54	8017.800	-10.54
	27	6306.750	6264.900	-0.66	5408.050	-14.25
	32	4342.850	4304.850	-0.88	3713.880	-14.48
**(640×480)**	37	2959.750	2924.300	-1.20	2475.430	-16.36
	Average results					
	QP	-	-	-0.82	-	-13.90

As shown in Table 2, compared with the H.265/HEVC algorithm for different ultrasound video resolutions (560×416 and 640×480), the proposed scheme can obtain 15.35% and 13.90% coding bit-rate savings on average, respectively, whereas the algorithm in [6] only reduced 0.90% and 0.82% of coding bit-rate on average.

**Fig 6:**
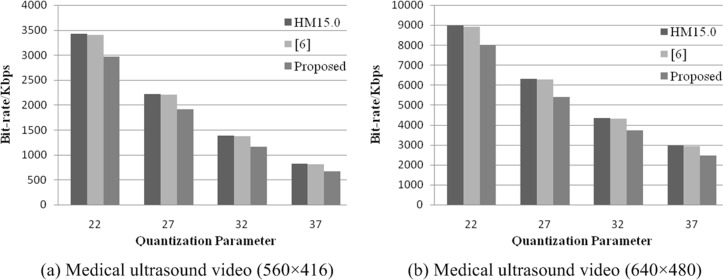
Comparison of the coding bit-rate.

In addition, to intuitively express the bit-rate saving reduction, the histogram of the bit-rate consumption is described in Fig 6. As shown in Fig 6, the proposed method enhances the advantage of low bit-rate coding compared to H.265/HEVC and [6]. The quad-tree structure shaped ROI extraction strategy is the precondition, and the otherness coding strategy for ROIs and non-ROIs is essential to improving the compression efficiency in medical video compression.

#### b) In terms of R-D performance

With the purpose of evaluating the R-D performance, the Bjøntegaard delta bit-rate (BDBR) was used to quantify the bit-rate gains achieved by H.265/HEVC. The Bjøntegaard delta peak signal-to-noise rate (BDPSNR) was used to measure the objective quality improvements of the encoded medical video [16]. Table 3 shows a comparison between the DBDR and BDPSNR results in [6] and the proposed method with H.265/HEVC.

**Table 3:** Comparison of BDBR and BDPSNR with the H.265/HEVC standard.

Sequence	[6]		Proposed	
(Resolution)	BDBR(%)	BDPSNR(dB)	BDBR(%)	BDPSNR(dB)
**Ultrasound video**	-0.59	0.04	-14.20	1.01
**(560×416)**				
**Ultrasound video**	-0.28	0.03	-12.83	1.32
**(640×480)**				

As shown in Table 3, the modified algorithm in [6] achieves an average bit-rate gain of 0.41% compared to the H.265/HEVC standard, while the average bit-rate saving is 13.5% in the proposed method. Furthermore, the proposed method can obtain an average PSNR gain of 1.16 dB compared to H.265/HEVC when providing an equivalent coding bit-rate, while it provided an average value of 0.035 dB in [6]. Therefore, the proposed method is able to achieve a better encoding performance improvement as quantified by BDBR and BDPSNR.

**Fig 7:**
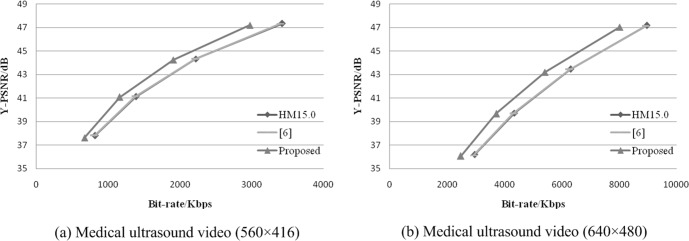
Comparison of the R-D performance.

For intuitional illustration purposes, the R-D performance curves of the three algorithms (the H.265/HEVC standard algorithm, the modified algorithm in [6], and the proposed algorithm) are shown at the different resolutions of 560×416 and 640×480, in Fig 7.

It can be observed that the R-D performance of the proposed algorithm outperforms H.265/HEVC and [6] in Fig 7. The coding performance improvement is mainly derived from the use of the transform coefficient adjustment and QP selection strategy in the hierarchical coding process.

#### c) In terms of visual quality

The structural similarity index measurement (SSIM) [17] is a widely used video quality assessment metric with a high consistency of subjective human visual systems. Here, SSIM was employed for a PSNR replacement to evaluate the visual quality of both the entire video and the ROI-layer video.

As shown in Table 4, for the full-length medical videos, the improvement of SSIM indexes are 0.0012 and 0.0017 on average with different resolutions of 560×416 and 640×480, respectively. The corresponding increases are 0.0016 and 0.0026 for ROI-layer videos, respectively. Although the SSIM gains are not significant from the perspective of the order of magnitude, the positive effects can be enhanced with the increase of QP values. This means that better SSIM gains can be obtained in low bit-rate applications in the medical video compression domain. The important reason for visual quality improvement is the proposal of the adaptive transform coefficient adjustment strategy in the hierarchical coding process. On this basis, the proposed method can provide better contrast for the human visual system compared to H.265/HEVC by enhancing the transform coefficients in ROIs but weakening the transform coefficients in non-ROIs.

**Table 4:** Comparison of SSIM with H.265/HEVC standard.

Sequence	QP	HM15.0	Proposed		HM15.0 (ROI)	Proposed (ROI)	
(Resolution)							
		SSIM*	SSIM*	△SSIM	SSIM*	SSIM*	△SSIM-ROI
**Ultrasound video**	22	0.9920	0.9922	0.0002	0.9922	0.9927	0.0005
	27	0.9850	0.9855	0.0005	0.9850	0.9859	0.0009
	32	0.9706	0.9717	0.0011	0.9696	0.9710	0.0017
	37	0.9426	0.9454	0.0028	0.9406	0.9441	0.0035
**(560×416)**							
	Average results						
	QP	-	-	0.0012	-	-	0.0016
**Ultrasound video**	22	0.9878	0.9880	0.0002	0.9868	0.9873	0.0005
	27	0.9708	0.9718	0.0010	0.9690	0.9705	0.0015
	32	0.9185	0.9201	0.0016	0.9150	0.9180	0.0030
**(640×480)**	37	0.8540	0.8578	0.0038	0.8476	0.8533	0.0057
	Average results						
	QP	-	-	0.0017	-	-	0.0026

* SSIM = 1 indicates that the two videos involved in the evaluation are fully consistent.

In general, the extensive experimental data indicates that the proposed method can provide significant bit-rate reduction for medical ultrasound video compression with a favorable visual quality. Meanwhile, we note that the improvements and optimizations in the proposed method are internal to the H.265/HEVC encoder and are compatible with other pre-process algorithms, such as the filtering process in [7]. Thus, the coding performance can be further improved by adding a medical video pre-processing step before the coding framework.

## V. Conclusions

A human visual selective attention mechanism has resulted in growing demand for ROI-based HEVC. For the purposes of solving low-efficiency problems in recent medical communication systems represented by the JPEG or JPEG2000 coding standard, innovative studies regarding d-ROI-based hierarchical coding strategy are presented in this paper for medical ultrasound video. Initially, ROIs were accurately obtained by using textural information as the mainly visual feature, which can improve the applicability of ROI extraction results compared to traditionally shaped ROIs. Thereafter, according to the ROI extraction results, the otherness coding process was implemented by transform coefficient adjustment and QP selection. Experimental results showed that the proposed method achieves 13.52% bit-rate savings on average and guarantees acceptable visual quality for clinical application compared to H.265/HEVC (HM15.0). The proposed method can satisfy the requirement of real-time and high-resolution compression in modern medical communication systems. The enhancement of the adaptability for QP selection processing will be studied in future work.
